# Communicating with parents of obese children: which channels are most effective?

**DOI:** 10.1111/hex.12463

**Published:** 2016-04-28

**Authors:** Melanie Randle, Anthony D. Okely, Sara Dolnicar

**Affiliations:** ^1^ School of Management, Operations and MarketingFaculty of BusinessAustralian Health Services Research InstituteUniversity of WollongongWollongongNSWAustralia; ^2^ Early Start Institute, School of EducationFaculty of Social SciencesUniversity of WollongongWollongongNSWAustralia; ^3^ UQ Business SchoolUniversity of QueenslandBrisbaneQldAustralia

**Keywords:** child obesity, parents, public communications, segmentation, social marketing

## Abstract

**Background:**

One of the strategies proven most successful in curbing rising rates of childhood obesity involves targeting parents as agents of change. Prior studies have focused on *what* messages to communicate, but few have investigated *how* they should be communicated.

**Objective:**

To identify the channels most effective for communicating with parents of overweight and obese children and understand whether their use of parenting information sources differs from others in the community.

**Design/setting:**

This study utilizes data from the Longitudinal Study of Australian Children (LSAC). Families were included if weight and height information was available for parents and children at three data collection points: Waves 1, 2 and 4 (collected 2004, 2006 and 2010, respectively, *n* = 5107).

**Analysis:**

*A priori* and *a posteriori* segmentation methods identified groups of parents that were similar in the sources used to obtain information about parenting, and examined whether some segments were more likely to have obese children.

**Results:**

Four segments were identified that differed in their information source use: the ‘personal networks’, ‘books’, ‘official sources’ and ‘mixed approach’ segments. The ‘official sources’ and ‘mixed approach’ segments were most likely to have obese children, and they used doctors, government/community organizations and friends to obtain information on parenting. These segments were also less educated and had lower employment.

**Conclusions:**

Messages are most likely to reach families with obese children if communicated through doctors, government publications and community organizations. Further, messages targeting social groupings of parents will leverage the power of advice from friends, which is another valuable information source for this group.

## Introduction

Worldwide obesity has doubled in the past 30 years, and it is now considered one of the most serious public health challenges of the 21st century.^1^ Of particular concern is the rise in rates of overweight and obesity amongst children and the associated serious physical, mental and emotional health consequences. Figures reveal that in the United States, one‐fifth of children are currently overweight or obese,^2^ and this rate is even higher in Australia, where the number rises to one in four.^3^ Contrary to historic trends, the rise in childhood obesity could lead to younger generations having lower life expectancies than their parents.^4^

Children who are obese suffer from a range of adverse health outcomes. They are more likely to develop type 2 diabetes, non‐alcoholic fatty liver disease, orthopaedic complications and psychological problems, and are less socially accepted by their peers.^5^ Obesity tracks strongly from childhood to adulthood, with adults who were obese as children likely to have higher levels of obesity and be more severely affected by the diseases associated with obesity.^6^

In response to this issue, governments around the world have developed and implemented a range of policies and programmes to curb the rise in rates of childhood obesity. The effectiveness of these initiatives has been systematically reviewed,^7^, ^8^, ^9^ and one of the strategies identified as most successful is targeting parents as agents of change.^10^, ^11^, ^12^ Findings from both marketing and health research indicate that parents are the key determinants and agents of change in children's eating and exercising behaviours.^11^, ^13^, ^14^, ^15^ If this is the case, it is also critical to learn how to communicate most efficiently with parents of young children, which has not been done previously. While some research has been conducted on the style of communication that might be most effective for health messages,^16^ little has been done on the effectiveness of different channels of communication. Therein lays the main contribution of this study, which directly addresses calls to identify ways of effectively communicating with different groups within the population.^17^, ^18^

Marketing theory postulates that marketing strategies are more effective when targeted towards specific groups in the population. Targeting includes identifying groups of consumers (referred to as market segments) who are similar to one another in some way and which makes them distinctly different from other groups of consumers.^19^ These similarities – whether they be life cycle stage, needs, interests or even socio‐demographic characteristics – enable marketers to develop customized products and communications targeted towards each of the segments. Targeting includes the nature of the message communicated, but also the communication channel chosen.

Hitherto, the focus regarding communicating with parents about childhood obesity has been on *what* to communicate; that is, what message content is likely to resonate with parents and influence their parenting behaviour in a positive manner. Far less attention has been paid to *how* these messages should be delivered; that is, the communications channels (or, from the parent's perspective, information sources) which are most likely to ensure the messages actually reach parents as intended.^20^ There is little general knowledge about the information sources utilized by parents to access information relating to parenting and health for their family. Furthermore, given the identified relationship between sociocultural background^21^ and child obesity, it is currently unknown whether parents' socio‐demographic characteristics, or the extent of their own overweight and obesity status, and that of their children, are related to where this information is sourced.

This study aims to identify the information channels most likely to reach parents of overweight children, and whether this differs for particular groups within the community. The specific research questions are as follows:

1. What are the main information sources used by parents to obtain information regarding parenting?
2. Do segments of the public exist which differ in their patterns of information source use regarding parenting, and if so, do they also differ in terms of socio‐demographic characteristics and weight status?

## Methods

### Data

Data were taken from the Longitudinal Study of Australian Children (LSAC, http://www.growingupinaustralia.gov.au), a nationally representative study funded by the Australian Department of Families, Housing, Community Services and Indigenous Affairs. The study collects information relating to the social, economic and cultural factors that affect the adjustment and well‐being of children, for the purpose of informing public policy. For each child involved in the study, a multimethod data collection strategy is utilized, which includes self‐completion questionnaires from both parents and in‐depth (approximately 40 min) interviews with the child's primary caregiver.^22^ Measurement instruments, including questionnaires, were developed following a methodical process based on several principals which guided the selection of (i) theoretical constructs (for example, explanatory power, population relevance, perceived importance to policy) and (ii) items and measurement scales (for example, established validity and reliability, acceptability to participants, comparability with other national and international studies).^23^

The LSAC includes a nationally representative sample of Australian children who were aged 3–15 months (cohort 1) and 4–5 years (cohort 2) at the start of the study in 2004. Children of appropriate ages were randomly selected from the national Medicare enrolment database, and invitations to participate were sent to the relevant Medicare cardholder who could indicate their consent or refusal to participate in the study. The LSAC collects information on a broad range of topics related to the child's development and environment, including, for example, the child's social and emotional development, health status and related behaviours, learning environment and outcomes, family and parenting environment, education and other relationships (details of measures are available at http://www.growingupinaustralia.gov.au). Specifically in relation to this study, information regarding weight status was collected in Waves 1, 2 and 4 of the study when the child was aged 0–1, 2–3 and 6–7, respectively (the term ‘waves’ refers to the series of data collection points within a longitudinal study). Therefore, families included in these analyses were those for whom this information was available at the corresponding data collection time points in 2004, 2006 and 2010 (*n* = 5107).

### Measures

Questions were answered by the person who knew most about the child (referred to as Parent 1) and the other parent of the child or partner of Parent 1 (referred to as Parent 2).

#### Information channel use

Parents were asked ‘(Apart from your partner) What are your three most important sources of information about parenting or caring for your child?’ Response options can be found in Table 3. Respondents could nominate up to three answers from the list.

#### Child weight status

To measure perceived child weight status parents were asked ‘Do you think your child is…’ and could finish the sentence by choosing ‘underweight’, ‘normal weight’, ‘somewhat overweight’, ‘very overweight’ or ‘don't know’. To measure concern about their child's weight, parents were asked ‘How concerned are you about your child's weight at the moment?’ and could answer ‘not at all’, ‘a little’, ‘moderately’ or ‘very’. To measure actual weight status, the trained interviewer weighed each child and measured their height using a portable stadiometer. The children were classified according to the International Obesity Taskforce (IOTF) cut points^24^ and as underweight using cut points derived from comparable methods.^25^

#### Parent weight status

Parents were asked how tall they were and how much they weighed. Weight status categories of parents were based on BMI values and can be found in Table 3.

#### Socio‐demographic characteristics

Parents were asked about their employment status, sources of income, perceived financial status, level of school completion, highest educational qualification, if they were of Aboriginal or Torres Strait Islander origin, and any existing medical conditions or disabilities using standardized questions.^26^ Response options can be found in Table 3.

### Analysis

Data were analysed under three different assumptions. The first analysis assumed that all parents used the same communication channels, and could thus be treated as one homogeneous group. Simple percentage distributions revealed how frequently parents used each channel for practical advice – including parenting advice – across all parents.

The second analysis, known as *a priori* 27 or commonsense segmentation,^28^ uses the weight status of the child as the segmentation criterion. The assumption therefore is that parents of children who (i) have never been overweight or obese; (ii) have been overweight/obese at one time point in the study; and (iii) have been overweight/obese across all time points in the study will differ systematically in the information sources they used. Chi‐square tests determined whether information source use differed across the three groups.

Finally, the third analysis approached the research problem from an entirely different perspective. We used an approach known as *post hoc*,^29^ *a posteriori* 27 or data‐driven^28^ segmentation, to identify groups of parents who shared similar patterns of information source used to obtain information about parenting. This approach is effective for identifying groups of parents that have characteristics in common which are not immediately observable.

For a sample size of 5107, up to 12 variables (information sources) can be used to conduct data‐driven segmentation.^30^ We therefore eliminated the ‘other’ variable as well as the ‘neighbours’ variable which showed the highest redundancy with another item, ‘friends’.

An algorithm referred to as topology representing networks^31^ was used because it outperforms most other cluster algorithms in extensive simulations with artificial data sets.^32^ To determine the number of segments, 20 repeated calculations were computed with segments between 2 and 10 (total 180 computations) to examine the data structure and select the most stable number of segments.^33^ Each repeated computation was based on a different bootstrap sample of the original data. The four‐segment solution was found to have the best properties and was thus chosen for further analysis. Differences in segment profiles were tested using analysis of variance for metric variables and chi‐square tests for nominal and ordinal variables.

## Results

### Communication channel use across all parents

Simple percentage distributions provide insight regarding the parenting information sources most frequently used across all parents and are shown in Table 1. Non‐resident family members were most commonly used (nominated by 77% of parents), followed by friends (61%), doctors (39%) and books, newspapers and magazines (30%). Least frequently used sources of information about parenting were teachers (1%) and priests or religious leaders (2%). These results indicate that, across all Australian parents generally, close social networks were used most commonly when parents sought information about parenting. This raises the question of the extent to which public authorities can actually influence such information sources, and suggests that further investigation is required to better understand the potential for public communication campaigns to reach and influence parents of children who are overweight or obese.

**Table 1:** Use of information sources to obtain information on parenting or caring for a child (total sample)

Information source	Percentage of sample
Family members not living with you	77
Friends	61
Doctors	39
Books, newspapers or magazines	30
Government, community or welfare organizations	24
Other professionals	16
Other family members living with you	6
Neighbours	5
Internet	5
Telephone services	3
Television or videos	3
Other	2
Priests or religious leaders	2
Teachers	1

### *A priori* segmentation

Families were grouped into three *a priori* segments: those including children who (i) were *never* overweight or obese during the study; (ii) were overweight or obese at *one* time point in the study; and (iii) were overweight or obese across *all* time points in the study.

Results are shown in Table 2, presented in order of the size of differences between segments. The only borderline statistically significant difference is the use of books, where families with overweight or obese children in both waves were the least likely to use books as an information source. For all other types of information sources, no significant differences were found between the three groups of parents.

**Table 2:** *A priori* segment differences in use of information sources by the number of times the weight status of the child was overweight or obese

	Times the weight status of child reported as overweight or obese			
	None (*n* = 2817)	Once (*n* = 757)	Twice (*n* = 552)	*P*‐value
Books, newspapers or magazines	32	32	27	0.051
Family members not living with you	78	76	78	0.301
Friends	63	63	60	0.311
Internet	5	6	5	0.223
Doctors	37	39	40	0.341
Neighbours	5	5	7	0.172
Other professionals	16	17	16	0.596
Government, community or welfare organizations	25	24	24	0.539
Telephone services	2	3	2	0.451
Television or videos	2	3	4	0.242
Other family members living with you	4	5	6	0.492
Other	2	2	1	0.660
Teachers	1	1	1	0.828
Priests or religious leaders	2	2	2	0.891

### Data‐driven segmentation

**Figure 1:**
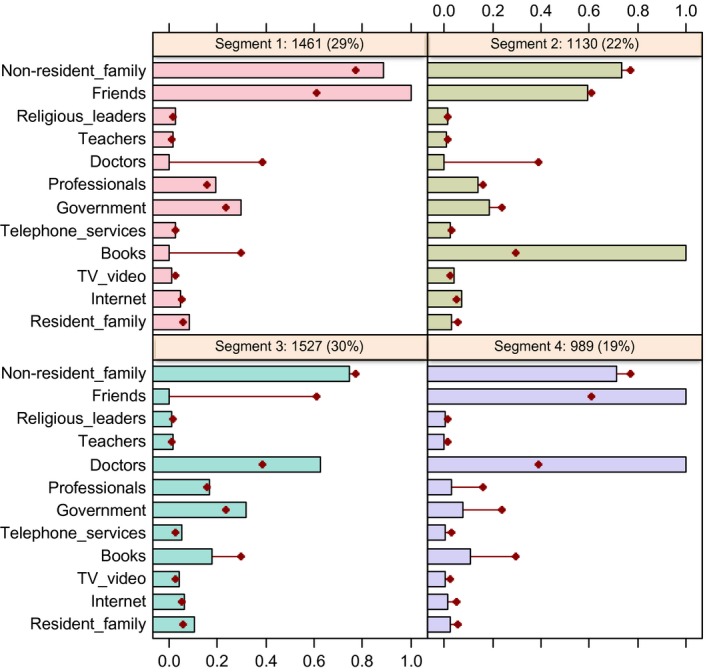
Information sources used by each segment compared to the average use across the population.

The four‐segment segmentation solution resulted in groups who used information sources in terms of the patterns depicted in Fig. 1. The four quadrants in Fig. 1 represent the four segments that emerged from the segmentation analysis. The shaded horizontal bars in each quadrant represent the percentage of parents in *that segment* who stated that they used each information source. The horizontal line with the dot at the end represents the percentage of parents in the *total sample* who use that information source. Distinguishing characteristics of a particular segment can be identified by comparing the length of the bar to the position of the respective dot. Where there is a large difference between the bar and the dot, this can be interpreted as being an information source that was used either substantially more or less often by that segment than by the overall sample of parents.

Segment 1 includes just over one‐quarter of the sample (29%), and they reported relying more heavily than the total sample on their personal network of friends and non‐resident family for parenting information. Because of this, they have been labelled the ‘personal network’ segment. They also used other professionals and information provided by the government, community and welfare organizations slightly more frequently than the total population.

Segment 2 contains slightly less than one‐quarter of the sample (22% of parents), and were more likely to read books, newspapers and magazines to obtain parenting information. This was the only information source used by this segment more frequently than the other segments (more than twice as often), and it has therefore been labelled the ‘books’ segment.

Segment 3 includes 30% of parents, which were those more likely than average to use doctors and government, community or welfare organizations to find information. This segment showed very little reliance on personal sources such as friends for information, but instead relied on official sources such as doctors and government. It has therefore been labelled the ‘official sources’ segment.

Finally, segment 4 includes around one‐fifth of parents (19%) who used both their personal networks (friends) plus official sources (doctors) more often than average to access parenting information. Because of this, they have been labelled the ‘mixed approach’ segment. These results, and the deviation of many of the bars across the segments from the average usage levels indicated by the dots, suggest that heterogeneity does exist among parents regarding their sources of parenting information. So, for example, while family and friends played a major role for some, they were not equally important to all segments.

To investigate the differences between segments further, Table 3 reports differences between the segments in the other family characteristics measured. Families in the ‘books’ segment were least likely to have overweight parents or children, with over 70% of children being in the normal weight range at each wave of data collection, which was either the highest or equal‐highest percentage of all the segments. The ‘books’ segment also had the highest levels of parent education (45% of mothers and 34% of fathers had completed a bachelors degree or higher) and employment (55% of mothers and 95% of fathers were employed).

**Table 3:** Additional segment differences (in percentage of parents within the segment)

Segment name	Segment				*P*‐value
	1	2	3	4	
	Personal network	Books	Official sources	Mixed approach	
Indigenous status					
Yes, Aboriginal	4.1	1.2	5.8	3.1	< 0.0001
Medical condition/s					
Yes	4.9	3.4	7.3	6.4	< 0.0001
Employment status – parent 1					
Employed	52.2	55.3	45.1	46.6	< 0.0001
Unemployed	2.5	2.9	4.0	3.4	
Not in labour force	45.3	41.7	50.9	50.0	
Employment status – parent 2					
Employed	95.0	95.0	90.7	93.3	< 0.0001
Unemployed	2.0	1.9	3.9	3.0	
Not in labour force	3.0	3.0	5.4	3.6	
Main source of income					
Wages/salary	29.6	32.3	27.0	26.6	< 0.0001
Business/partnership	6.4	6.9	5.2	5.4	
Rental property	2.1	1.9	1.1	1.6	
Dividends/interest	2.7	3.3	1.6	2.6	
Govt pension/allowance	49.4	43.9	54.9	55.2	
Child support/maintenance	0.9	0.8	1.1	1.1	
Superannuation/annuity	0.1	0.0	0.0	0.0	
Workers' compensation	0.1	0.1	0.1	0.1	
Family's financial status					
Prosperous	1.4	2.3	1.2	1.7	< 0.0001
Just getting along	32.3	29.4	37.7	33.6	
Very poor	0.6	0.3	0.7	0.8	
School completion – mother					
Year 12 or equivalent	70.3	78.1	58.3	61.7	< 0.0001
Year 11 or equivalent	11.4	7.5	12.6	13.1	
Year 10 or equivalent	14.2	11.9	20.8	20.2	
Year 9 or equivalent	2.5	1.3	5.3	3.3	
Year 8 or below	1.5	0.9	2.6	1.2	
Never attended school	0.0	0.0	0.2	0.1	
Highest qualification– mother					
Postgraduate degree	6.2	11.2	5.8	5.7	< 0.0001
Graduate diploma/certificate	6.8	7.3	5.8	5.0	
Bachelors degree	21.4	26.7	14.1	17.0	
Advanced diploma/diploma	11.1	9.9	8.8	9.0	
Certificate	24.3	20.9	26.7	25.9	
School completion – father					
Year 12 or equivalent	54.0	62.1	46.0	47.1	< 0.0001
Year 11 or equivalent	10.0	8.8	10.4	12.0	
Year 10 or equivalent	20.9	21.3	22.2	22.8	
Year 9 or equivalent	3.2	2.9	3.6	3.7	
Year 8 or below	1.1	0.5	2.3	2.3	
Never attended school	0.0	0.0	0.1	0.0	
Still at school	0.1	0.0	0.0	0.0	
Wave 1 data collection (2004)					
Postgraduate degree	5.9	9.6	4.6	5.2	< 0.0001
Graduate diploma/certificate	5.3	5.6	5.2	3.6	
Bachelors degree	15.3	18.6	10.8	15.3
Advanced diploma/diploma	7.3	9.4	6.4	7.0
Certificate	33.3	30.2	33.3	33.2
Wave 1 data collection (2004)					
Weight status – parent 1					
Normal	42.5	51.6	42.8	43.1	< 0.0001
Overweight	26.8	24.0	25.0	26.5	
Obese class 1	13.9	8.0	11.8	13.0	
Obese class 2	4.0	3.1	4.7	4.9	
Extreme obesity	1.8	1.2	2.7	2.4	
Weight status – parent 2					
Normal	31.5	33.4	31.8	29.8	0.048
Overweight	46.8	50.2	45.2	49.5	
Obese class 1	16.1	10.8	16.2	14.8	
Obese class 2	3.0	2.0	3.1	2.4	
Extreme obesity	0.6	0.7	1.1	1.5	
Wave 2 data collection (2006)					
Concern about child's weight					
Not at all	86.5	85.7	84.4	86.7	0.297
A little	11.1	12.0	11.6	10.8	
Moderately	1.6	1.7	2.7	1.8	
Very	0.5	0.7	1.2	0.6	
Judgment about child's weight					
Underweight	5.5	7.7	7.2	6.8	0.698
Normal	91.6	89.6	90.1	90.3	
Somewhat overweight	2.6	2.5	2.4	2.8	
Very overweight	0.1	0.1	0.0	0.0	
Child's weight status					
Normal	73.1	74.5	70.8	69.2	0.011
Overweight	17.9	16.9	18.5	19.6	
Obese	4.6	2.9	5.5	5.1	
Weight status – parent 1					
Normal	47.7	55.4	47.2	47.2	< 0.0001
Overweight	25.3	21.3	24.5	24.3	
Obese class 1	11.3	7.8	10.2	13.4	
Obese class 2	4.1	2.4	3.2	3.6	
Extreme obesity	1.4	1.0	3.0	1.5	
Weight status – parent 2					
Normal	32.4	31.8	29.3	24.3	0.048
Overweight	47.3	50.5	49.5	51.4	
Obese class 1	16.2	12.0	16.2	18.1	
Obese class 2	2.2	2.9	2.4	2.8	
Extreme obesity	0.8	0.7	0.8	1.1	
Wave 4 data collection (2010)					
Concern about child's weight					
Not at all	85.9	84.9	83.9	84.5	0.016
A little	11.1	12.8	12.9	11.8	
Moderately	1.8	2.2	2.2	3.3	
Very	1.2	0.1	1.0	0.4	
Judgment about child's weight					
Underweight	5.9	7.1	8.0	9.2	0.278
Normal	90.9	90.4	88.9	88.3	
Somewhat overweight	3.0	2.5	2.9	2.5	
Very overweight	0.2	0.0	0.1	0.0	
Child's weight status					
Normal	71.7	71.0	69.9	71.4	0.778
Overweight	17.0	17.1	17.5	17.4	
Obese	5.5	4.8	6.5	5.0	
Weight status – parent 1					
Underweight	8.2	10.0	10.5	7.7	< 0.0001
Normal	44.1	53.3	43.0	43.3	
Overweight	28.4	23.1	27.1	26.9	
Obese class 1	11.5	9.1	11.8	13.6	
Obese class 2	5.3	3.0	4.2	5.6	
Extreme obesity	2.4	1.4	3.5	2.9	
Weight status – parent 2					
Underweight	1.8	1.8	2.0	2.2	0.065
Normal	30.2	34.0	28.2	25.3	
Overweight	47.5	48.1	48.3	49.9	
Obese class 1	15.8	13.5	16.6	17.4	
Obese class 2	4.2	1.9	3.4	4.4	
Extreme obesity	0.5	0.7	1.5	0.8	

The ‘official sources’ segment (which used doctors and government, community and welfare organizations as primary sources of information) and the ‘mixed approach’ segment (which used a mix of friends and doctors to obtain parenting information) were the two groups most likely to have overweight or obese children (an average of 24% of children in both segments were overweight or obese across all waves). These two segments were also most likely to have one parent who was obese (average of 21% of families in the ‘official sources’ had at least one obese parent across all waves, while this figure was 19% for the ‘mixed approach’ segment). However, these two segments were no more or only slightly more concerned about their child's weight than parents in other segments, with no significant differences found in levels of concern at wave 2, and only two percentage points difference between all segments in wave 4 (range 84–86% not at all concerned about their child's weight). Further, the ‘official sources’ and ‘mixed approach’ segments were not more likely to judge their child as being overweight, with no significant differences found between segments. These segments had the lowest levels of education (26% of mothers and 21% of fathers in the ‘official sources’ segment and 28% of mothers and 24% of fathers in the ‘mixed approach’ segment had completed a bachelors degree or higher). This was also the case for employment (45% of mothers and 91% of fathers in the ‘official sources’ segment and 47% of mothers and 93% of fathers in the ‘mixed approach’ segment were employed). The ‘official sources’ and ‘mixed approach’ segments were also more likely to rely on government pensions or allowances than the ‘personal network’ and ‘books’ segments; with over half of families (55% of families in both segments) stating that this was their main source of income. In addition, the ‘official sources’ segment was also the group most likely to report being of Aboriginal or Torres Strait Island origin (6%) and suffering from existing medical conditions (7%).

## Discussion

It has been established that targeting parents as agents of change is critical in stemming rising rates of childhood obesity.^10^, ^11^, ^12^ Central to this proposition is identifying the most effective ways of communicating healthy eating and physical activity messages to parents, especially of overweight and obese children.^34^ This study uses empirical data from a nationally representative longitudinal study of Australian families to investigate which channels are most effective for public communications with parents about childhood overweight and obesity, and whether this differs for particular groups within the community.

The key insights emerging from this study are twofold. First, segmenting parents based on the weight status of their child failed to reveal significant differences in the information sources utilized. This was evidenced by the results of the first *a priori* segmentation analysis, which did not identify any significant differences in the way families with overweight and obese children obtain information about parenting when compared to families with children of healthy weight. However, the second and potentially more valuable finding from this study is that by constructing segments based on their patterns of information source use, groups were identified which differed significantly in terms of children's weight status and other socio‐demographic characteristics. This was evidenced from the results of the second analysis which utilized *a posteriori* segmentation techniques. The two segments of families most likely to experience child and parent overweight and obesity were the ‘official sources’ and ‘mixed approach’ segments, which relied more on doctors, the government and other community organizations and friends for information than did the other parent segments. From a public communications perspective, this is encouraging because authorities can, to some degree, influence the information available from doctors, government departments and other community and welfare groups. Results here indicate that information communicated through these channels is most likely to reach the target audience of families with overweight and obese parents and children.

Results also show that parents do rely on different sources of information, depending on their socio‐demographic characteristics, a finding consistent with previous research in the area of health communications.^35^ Parents in the ‘official sources’ and ‘mixed approach’ segments were more likely to be from lower socio‐economic backgrounds, including lower levels of education. Public communications regarding child health should target general practices and use doctors and other medical staff to communicate key messages in formats suitable for less‐educated individuals, which may include both written and verbal formats. For the ‘official sources’ segment, this strategy is likely to be particularly effective, given that they also suffer more from medical conditions and are therefore likely be engaging with medical centres more often.

For the ‘mixed approach’ segment, friends were an additional valuable source of information. Recent research has shown that obesity is strongly associated with social networks among adults.^36^ Future communications targeted at the ‘mixed approach’ segment should target such social networks, which may include, for example, mothers groups, churches, workplaces, schools or any environment where parents are likely to interact socially.

Theoretically, this study provides evidence that within the general population, groups of families exist that engage with different sources of information about parenting. Practically, these findings suggest that generic campaigns communicated to the general population may, to a large extent, be reaching families for whom the message (in this case relating to child overweight and obesity) has no immediate relevance or use. Being able to target health‐related messages directly at those groups who are likely to benefit most from the information means that government and other public authorities can achieve maximum efficiency and effectiveness from the public funds spent on such communications campaigns, which ultimately benefits society as a whole.

A limitation of this study is that parents answered questions about sources of parenting and childcare information generally, not specifically child‐health information. Although it can be assumed that health‐related parenting information is a subset of general parenting information, it would be useful to replicate this study with data specifically focusing on health‐related information sources.

## Conclusion

Within the general population, different types of families rely on different sources of information about parenting. Families that include parents and children who are overweight or obese are more likely than non‐overweight families to rely on doctors, the government and other community organizations and friends for information about parenting. Utilizing these channels for health‐related communications increases the likelihood that key messages will reach families who stand to benefit most from the information, and also improves the efficiency with which public funds for health communications are spent.
